# Supplementary material for: Are carbon emissions trading and green financial instruments synergistic? -Comprehensive quantitative research based on content analysis
Source: PLoS One. 2024 Mar 7;19(3):e0298601. doi: 10.1371/journal.pone.0298601 (PMC10919601; doi:10.1371/journal.pone.0298601)
Supplement: S1 Data — (ZIP) [file pone.0298601.s001.zip › Data/地级市Z_F工作报告.zip!/┴╔─■/░░╔╜╩╨/└┤╫╘╣1⁄2╓┌║┼í╛┬φ┐╦╩2╛▌═°í┐_2013.docx]

各位代表：

现在，我代表市第十四届人民政府向大会报告工作，请予审议，并请市政协各位委员提出意见。

一、过去五年主要成就及2012年工作回顾

市第十四届人民政府在省委、省政府和市委的正确领导下，在市人大、市政协的监督支持下，团结带领全市人民，全面实现了第十四届人民代表大会确定的各项任务目标。

――综合实力全面提升。五年来，全市地区生产总值年均增长15.8%，公共财政预算收入年均增长19%，全社会固定资产投资年均增长29.5%，社会消费品零售总额年均增长18.6%。城市综合实力明显增强。

――经济结构加速优化。全市规模以上工业增加值年均增长15%；第三产业增加值年均增长15.7%，占全市经济比重由40.4%提升到42.4%；设施农业面积由16.9%提升到41.6%。民营经济比重达到68%。科技对经济增长贡献份额达到61%。被评为全国科技进步先进市。

――改革开放不断深化。国有企业改革深入推进，非公有制经济较快发展。对外开放不断扩大，实际利用外资和引进域外资金分别年均增长34%。被评为全国投资环境百佳城市。

――城乡环境明显改善。城市发展空间有效拓展。新增城市主次干道328条，新建改造供水管网312公里，新铺设燃气管网165公里，新增城区供暖面积1900万平方米。在全省率先实现全部行政村通柏油路、通客车。万元地区生产总值综合能耗年均下降5.5%。森林覆盖率由46.4%提高到48.1%。被评为全国创建文明城市先进市和国家森林城市。

――社会事业快速发展。在全国率先实行公立医院改革。在全省率先实现每个乡镇都有高标准九年一贯制（寄宿）学校，每个行政村都有“农家书屋”，每个社区（村）都有健身场所。职教城被确定为全国职业教育改革试点。建成市博物馆和文化艺术大厦。鞍山籍体育健儿在北京和伦敦奥运会上获得金牌、奖牌总数双列全省第一名。先后被评为全国文化体制改革先进市、全国人民防空先进市、全国计生综合改革示范市，蝉联全国双拥模范城市五连冠和全国无偿献血先进市五连冠。

――人民生活水平明显提高。城市居民人均可支配收入年均增长13.7%，农民人均纯收入年均增长15.1%。累计为群众办实事244件。城镇登记失业率控制在2%左右，被评为全国创业先进城市。棚户区加快改造，城市人均住房面积由22.4平方米增加到27.9平方米。在全省率先开展新型农村合作医疗和城镇居民社会养老保险，社会保障体系基本形成。社会管理创新深入推进，群众合理诉求妥善解决，社会综合治理、安全生产管理、食品药品管理进一步加强，社会保持和谐稳定。

各位代表，2012年面对国内外严峻复杂的经济形势，全市上下牢牢把握“稳中求进、稳中求快”总基调，全力稳增长，加速调结构，实现惠民生，经济呈现了企稳回升的良好态势。预计全年实现地区生产总值2687亿元，同比增长12%；公共财政预算收入234亿元，增长13%；全社会固定资产投资1740亿元，增长26%；社会消费品零售总额702亿元，增长16%；城市居民人均可支配收入24279元，增长14%；农民人均纯收入12818元，增长15%；万元地区生产总值综合能耗下降3.8%。

（一）全力稳增长，经济保持平稳较快发展

抓项目建设稳增长。开展项目建设年活动，滚动实施出让土地、招商引资、整治违章、加速审批、项目建设“五个百日行动”。全市在建项目2600项，总投资4663亿元。新开工项目1760项，总投资1380亿元。预计实现规模以上工业增加值1088亿元，增长13%；第三产业增加值1138亿元，增长13%。

抓招商引资稳增长。先后赴港澳台、珠三角、长三角等地开展主题招商。预计全年实际利用外资12.7亿美元，增长15%；引进域外资金1040亿元，增长30%；外贸出口23亿美元，增长15%。全年向上争取资金150亿元，金融机构新增本外币贷款175亿元。

抓扶持企业稳增长。出台一系列稳增长政策措施，帮助企业解决用地、用电、融资等生产经营中的突出问题，支持企业扩大生产、稳定经营。帮助海城、岫岩受灾企业恢复生产。

抓地企合作稳增长。支持鞍钢不锈钢复合板、特钢板和煤化工产业项目。积极为中冶集团、中钢集团、中船重工等央企上项目提供服务。鞍钢等中省直企业为鞍山稳增长做出了重大贡献。

（二）加速调结构，转变经济发展方式取得进展

工业结构不断优化。钢铁深加工、菱镁新材料、装备制造业、纺织服装业等特色产业进一步发展，精细化工、高端阀门、激光、能源电池等新兴产业不断壮大。全年新增规模以上工业企业80家，总数达到1167家。

现代服务业加速发展。充分发挥城区经济的支撑作用，出台了《加快发展现代服务业实施意见》。主城区商业综合体、品牌连锁经营、楼宇总部、金融服务、文化旅游等项目加速建设。全年新增限额以上服务业企业245家，总数达到2827家。

房地产业平稳发展。积极吸引国内外知名开发企业来鞍投资。通过举办房交会，出台惠民政策，刚需购买力得到较好释放。预计全年完成房地产投资额386.6亿元；实现商品房销售额308亿元，增长22%。

（三）立足统城乡，县域经济较快发展

预计全市县域地区生产总值增长21%，公共财政预算收入增长17.3%，固定资产投资增长27%，高于全市平均增长水平。

现代农业蓬勃发展。海城优质大蒜种植面积发展到5万亩，南果梨栽植面积发展到33万亩；台安鸭鹅饲养量和年加工能力达到1.5亿只，成为东北鸭鹅第一县；岫岩克服洪灾影响，食用菌产量恢复到40万吨。全市设施农业拉动农民人均增收6000元以上。海台岫三个农高区总面积217平方公里，引进十亿元以上农业产业化项目10个，总投资161亿元。

县域工业不断壮大。海城打造千亿菱镁新材料产业基地，62个入园项目加速建设。台安规划建设10平方公里彩涂板深加工产业基地，8个重点项目落地开工。岫岩规划建设大洋河临港产业区和雅河工业园，36个项目逐步落地。全市县域工业园区累计入驻亿元以上项目397个。

县域服务业加速集聚。海城西柳中国商贸城投入运营，南台箱包城配套加工园入驻企业26家。台安八角台新城入驻限额以上商业企业105家。岫岩玉皇山文化旅游区已有中国玉文化艺术园等4个重点项目开工建设。

（四）注重增活力，深化改革取得新成果

综合配套改革有序推进。利用沈阳经济区综合配套改革先行先试政策，全力推进新兴产业、现代服务业、循环经济、城市矿产资源利用、职业教育、社会管理创新等综合配套改革。鞍山高新区被确定为省级新兴产业示范区，汤岗子新城被确定为省级现代服务业试验区。鞍山经济开发区被商务部确定为国家级钢铁物流出口基地。

产业园区活力不断增强。完成激光产业园等5个新兴产业园区发展规划。出台了《汤岗子新城享受市级经济和部分社会管理权限的意见》。按照明确区划、制定规划、产业定位、政策扶持、优化机构、健全机制、完善设施、引进项目的发展思路，创新产业园区和新城新市镇体制机制。全年各园区新引进项目1500个，总投资1100亿元。

科技创新能力持续提高。出台了《鞍山市科学技术进步条例》。搭建了激光技术测试中心等7个公共科技服务平台。引进海外研发团队17个。申报国家高新技术企业8家。预计全年完成高新技术产品增加值334亿元，增长30%。

非公有制经济较快发展。出台了《鞍山市促进中小企业发展条例》。建立中小企业贷款工作联席会议制度。推进鞍山重矿股份成功上市。支持中小微企业发展，加速培育规模以上工业企业、限额以上商业企业、资质以上建筑企业和房地产开发企业。预计全年民营经济实现增加值1840亿元，增长20.3%。

（五）着力优环境，城乡面貌得到改善

新城建设加速推进。启动汤岗子新城主干路网、中央活力区、凤凰湖中央公园等基础设施和招商引资项目建设。达道湾新城一批品牌连锁商场、商务楼宇、体育场馆、职教设施和交通道路加速建设。海西新城主干路网和城市环路基本建成。

腾鳌新城路网及设施配套工程加速推进。析木新城纳入沈阳经济区新城建设规划。批准建设台安八角台新城和岫岩玉都新城。

城乡建设稳步推进。建国南路和杨柳河桥拓宽改造主体工程竣工通车；建国东路、千山东路、机场路等城市主次干道改造工程基本完成。沈西工业走廊出海通道、哈大高铁鞍山西客站竣工通车。腾鳌机场列入国家民航规划序列。建筑围挡、广告牌匾等城市设施管理水平明显提升。

生态环境逐步改善。推动实施青山、碧水、蓝天、净土工程。完成矿山环境治理1.4万亩，造林绿化4.4万亩，封育围栏150公里。辽河生态治理任务全面完成，大孤山等8座污水处理厂基本建成，国控断面达到地表水三类标准。羊耳峪垃圾处理场环境初步改善。6项空气污染减排项目较好完成，城区空气环境质量达标天数提高到350天。

（六）实现惠民生，社会事业全面发展

民生工程扎实推进。为城乡群众办的20件实事较好完成。全年实现城镇实名制就业13.3万人，城镇登记失业率下降到1.97%。城市居民医保最高报销限额由6万元提高到21万元，新型农村合作医疗最高报销限额由5万元提高到12万元。城乡最低生活保障标准分别提高15%和25%。居民消费价格指数涨幅低于全国全省平均水平。建成回迁住房281.5万平方米，3.1万户居民陆续回迁。建成各类保障性住房7509套。改造36所农村敬老院。帮助256户农村困难家庭解决住房。资助1046名困难家庭大学生。新增100辆城市公交车。全市安全生产事故起数下降50%。推进社会治安防控体系建设，严厉打击各类违法犯罪，人民群众安全感进一步增强。

社会事业不断进步。新建改造25所标准化公办幼儿园、10所中小学校和5所高中。县区普九全部达到“双高”水平。开展药品集中采购试点。为全市43万名老年人免费体检。奥体中心和市民健身中心基本建成，十二届全运会我市筹备工作全面展开。建成鞍山文化大市场。完成图书馆“一卡通”工程。启动有线电视数字化整转工作。市人口和家庭公共服务中心建成使用。妇女儿童、老年人、残疾人合法权益有效维护。民族宗教政策很好落实。气象灾害和地震监测达到省内一流水平。外事、侨务、对台、统计、史志、档案、人防、民兵预备役等工作取得新成绩。

灾后重建有序进行。“8.4”洪灾造成我市15万亩农作物绝产，7.2万栋大棚不同程度受灾。全市上下全力以赴抗洪救灾，较快完成交通、电力、通讯和饮水设施抢通工程，及时为受灾群众发放救灾物资，实现了大灾之后无疫情。入冬前1832户水毁房屋的受灾群众搬进新居。实施灾后恢复生产扶持政策，受灾企业基本恢复生产，受损大棚大部分已恢复。

（七）加快转职能，政府依法行政水平有所提高

自觉接受市人大及其常委会法律监督、工作监督和市政协民主监督。认真落实人大议案。全年办理人大代表建议、批评和意见127件，办理政协提案294件。提请审议地方性法规草案1件，制定和修正政府规章8件，受理行政复议案件45件。支持检法机关依法行使职权。加大审计监察工作力度，社会信用体系建设稳步推进。全省公共资源交易系统建设试点取得初步成效。建立健全了鼓励先进、奖优罚劣的责任目标体系。

各位代表，经过全市上下的共同努力，我们不仅战胜了经济发展中的困难和自然灾害的挑战，而且为建设富庶文明幸福新鞍山奠定了良好基础。这些成绩的取得，得益于市委的正确领导和市人大、市政协的监督支持，得益于全市各族人民的团结奋斗，得益于各县（市）区、开发区、各部门的辛勤工作，得益于以鞍钢为代表的各类企业的巨大贡献。在此，我谨代表市政府向为鞍山发展付出辛劳的各位代表和委员，向奋战在全市各条战线的广大干部和群众，向各民主党派、工商联、无党派人士、人民团体，向中省直单位、驻鞍部队、武警官兵，向关心支持鞍山发展的老领导、老同志，向所有为鞍山发展做出贡献的海内外朋友，表示衷心的感谢和崇高的敬意！

各位代表，在总结成绩的同时，我们也清醒地看到鞍山经济社会发展中还存在许多矛盾和问题。主要是经济发展下行趋势明显，运行中的困难依然很多；战略性牵动性重大项目较少，优势新兴产业比重较低；节能减排任务艰巨，环境保护压力巨大；新城新市镇建设步伐需要进一步加快；以改善民生为重点的社会事业任务繁重；软环境还需不断改善，政府自身建设仍需加强。这些问题需要我们今后采取有力措施，认真加以解决。

二、今后五年奋斗目标及2013年主要任务

今后五年是鞍山老工业基地全面振兴的决胜期和全面建成小康社会的关键期。我们将紧紧抓住沈阳经济区和沿海经济带双重叠加优势，推进鞍山发展取得新进步。今后五年工作指导思想是：以邓小平理论、“三个代表”重要思想和科学发展观为指导，全面贯彻落实党的十八大和省委十一届五次全会精神，按照市委提出的“三转型一创新”和三个“两手抓”的总体要求，坚持发展第一要义，全面深化改革开放，转变经济发展方式，创建全国文明城市，在稳增长中提升质量效益，在调结构中推动转型升级，在惠民生中实现生活富裕，聚精会神搞建设，一心一意谋发展，努力建设富庶文明幸福新鞍山。

今后五年奋斗目标是：努力实现地区生产总值、公共财政预算收入、全社会固定资产投资、社会消费品零售总额、城乡居民人均收入增长高于全省平均水平。继续打造世界级钢铁深加工产业基地、世界级菱镁新材料产业基地、世界级健康养生产业基地。加速提升辽宁中南部中心城市竞争力，率先实现全面建成小康社会的奋斗目标。

各位代表，2013年是全面贯彻落实十八大精神的开局之年，是实施“十二五”规划承前启后的关键一年，是为全面建成小康社会奠定基础的重要一年。我们应该看到，当前世界经济总体需求不足，改革开放瓶颈制约依然存在，发展仍然面临许多困难和挑战。我们更应看到，我国仍处于可以大有作为的重要战略机遇期，新型工业化、新型城镇化、区域一体化和资源市场化为我们提供了新的发展机遇，十八大提出的一系列新思想、新目标、新举措，更为经济社会发展提供了不竭动力。我们还应看到，经过多年持续不断的结构调整、城乡建设和改革开放，为我市持续协调发展奠定了良好基础。我们站到了更高的起点上，一定要理性认清形势，坚定发展信心，准确把握机遇，努力拼搏实干，全力推动鞍山老工业基地新的崛起！

2013年主要预期目标是：地区生产总值增长11%，公共财政预算收入增长12%，全社会固定资产投资增长20%，社会消费品零售总额增长16%，城市居民人均可支配收入增长12%，农民人均纯收入增长13%。

（一）进一步推进农业现代化，壮大县域经济

以产业化经营为重点，加快发展现代农业，提高农产品精深加工比重，加速县域工业园区、服务业集聚区和新城新市镇建设，带动县域经济继续在全市率先发展，成为鞍山经济发展的重要支撑。全年县域地区生产总值增长20%，公共财政预算收入增长18%。

大力推进农业产业化。加速海台岫三个农高区建设，重点推进江苏润恒农产品物流园、海城三星无公害设施农业等十亿元以上农业产业化大项目，全年新培育规模以上农产品深加工企业30家。加速岫岩国家级食用菌出口示范区建设。组建综合性农业社会化公共服务平台。积极发展农民专业合作社，引导农民以多种方式流转土地承包经营权，探索土地股份制经营。推进抵押性融资方式，扩大农业保险规模和品种，构建以企业投入为重点、财政投入为引导、农民投入为主体的农业多元化投入机制。

积极推进农业设施化。大力发展设施种植业、设施养殖业、设施林业和设施渔业。按照“一县一业”的总体要求，海城继续扩大优质大蒜和南果梨种植规模；台安鸭鹅养殖发展到2亿只；岫岩食用菌培植恢复到60万吨。加快实施大型泵站改造、节水滴灌、果树引水上山等水利化项目。推广水稻生产全程机械化。发展保护地农业、工厂化农业。

有效推进农业科技化。加强与大专院校、科研院所技术合作，促进农业科技成果转化应用。全年引进示范推广农业新品种100项、新技术50项、新装备20项。加强基层农业技术推广体系建设。加快农村实用人才和专业人才队伍建设，培育职业农民。

（二）进一步推进新型工业化，建设工业强市

以工业产业集群建设为重点，深入实施产业集群、企业升级、项目建设、企业并购、淘汰落后“五项工程”，让各类高新区、开发区和工业园区成为推进新型工业化的主战场。全年规模以上工业增加值增长11.5%。

大力发展产业集群。推动钢铁深加工、装备制造业、菱镁新材料、精细化工、高端阀门、激光科技、能源电池、健康制造、彩涂板、临港产业等优势产业集群发展。各类产业集群全年开工建设亿元以上项目238个，新增规模以上工业企业90家。

加速工业园区建设。明确工业园区主导产业和发展重点，突出专业性、集群化。完善工业园区交通、供电、供水等基础设施。加快研发、检测、人力、金融、总部、展销等公共平台建设。

支持鞍钢等骨干企业发展。促进鞍钢加速结构调整，发展非钢产业，建设钢铁物流平台，打赢扭亏增效攻坚战。支持各类企业开展合资合作、项目融资、品牌建设、并购重组，培育一批行业领军企业。推动中冶集团、中钢集团、中船重工等驻鞍企业做强做大。

提升创新驱动能力。支持企业实施技术改造、搬迁升级、专利申请、引进人才和海外研发团队。新建省级重点实验室和省级工程技术中心，新建公共服务平台和研发中心各10个，新增国家高新技术企业10家，力争全市高新技术产品增加值增长30%。

（三）进一步推进服务业集聚化，提升城区发展水平

以服务业集聚区建设为重点，大力发展现代服务业，让城区加速成为城市经济发展的主力军，发挥区域中心城市辐射带动作用。全年开工建设亿元以上服务业项目220个，第三产业增加值增长12%。

优先发展生产性服务业。重点建设达道湾钢铁物流交易中心、汤岗子健康产业基地、高新区激光科技城、立山商贸城、西柳服装城和南台箱包城。发展钢铁物流、健康服务、激光研发、服装销售等服务于优势工业产业集群的生产性服务业。

着力发展新兴服务业态。中心城区要在繁荣发展商贸服务业的基础上，加速发展以城市综合体、品牌连锁经营、电子商务、金融保险、文化创意、中介服务等新兴服务业态为主的楼宇经济，增强中心城区对全市经济的辐射带动能力。

加速发展文化旅游产业。重点建设千山风景名胜区、文化旅游开发区、玉佛苑文化旅游区、汤岗子温泉养生度假区、腾鳌温泉休闲度假区、白云山风景旅游区、辽河生态旅游区、玉皇山文化旅游区等文化旅游产业集聚区。发展温泉养生、休闲度假、文化娱乐、健康运动、乡村旅游等旅游度假产业。在旅游产业发展中促进文化产业大发展。

在今后一个时期的城区发展建设中，要特别突出做好创建全国文明城市工作。要建立健全创城工作的长效机制，通过创城带动城市亮化、绿化、净化、美化和文化水平的提高，把鞍山建设成为活力旺盛、生态宜居的现代中心城市。

（四）进一步推进新型城镇化，建设美丽鞍山

按照产城融合、区域一体、生态宜居的发展思路，科学编制新一轮鞍山城市总体规划，注重规划的程序化、法制化管理。以交通基础设施、生态环境和城市信息化建设为重点，加快提升新型城镇化水平。

加速推进新型城镇化。努力做大做强中心城市，重点建设铁东中央商务区、铁西高铁新区和东部万水河新区。以承办全运会项目为契机，全面推动城市建设和管理水平的优化提升。加速推进汤岗子新城、达道湾新城、海西新城、八角台新城、玉都新城、腾鳌新城、析木新城和牛庄新市镇建设，加快推动鞍海一体化发展。要因地制宜、合理布局，加快区域特色中心镇建设，充分发挥统筹城乡关键节点的作用。

积极发展房地产业。引进国内外知名房地产开发企业，提升商品房开发和销售水平。强化小区配套、环境改造和物业管理工作，创造良好的宜居环境。全年实现房地产投资额和商品房销售额均增长15%以上。在稳步发展住宅地产的同时，积极发展工业地产、商业地产、楼宇地产、旅游地产和文化地产等各类地产。

大力发展建筑产业。积极培育资质优、专业精的建筑企业，重点支持规模大、实力强的建筑企业做强做大，推进建筑企业向大型化、高端化和外向化发展。全年实现建筑业增加值增长30%以上。

加快交通等基础设施建设。加快完善环城兴业大道建设，实现城区与园区、城市与周边的有效衔接。加快汤岗子新城主干路网、中央活力区路网、凤凰湖中央公园、康复养生基地和中国国际商会健康产业园等基础设施建设。加速腾鳌机场民用化改造，建设机场盲降系统，开通鞍山至上海、广州等新航线。推进鞍海城际铁路和城市地铁、海岫铁路提级改造、沈盘货运铁路台安段、鞍台高速公路、高铁大街等重点项目建设，完善鞍山城乡交通能力。

加强生态环境建设。深入实施青山工程，完成城市生态园林规划，实施造林绿化、矿山绿化、墓园绿化和路网绿化，全年完成小开荒还林2万亩，超坡地造林2.2万亩，封育围栏1100公里，矿山生态环境治理1.1万亩，墓园绿化12.6万株。继续实施碧水工程，强化城市污水处理厂运行管理，新建汤岗子新城污水处理厂，推进辽河生态恢复工程，继续推动万水河、运粮河、杨柳河、海城河、小柳河、大洋河等城镇水系景观建设工程，加快推动万水河公园、莲花湖公园、文化岛公园、激光主题公园、凤凰湖公园、高铁广场公园、奥体广场公园、营城子公园等水系公园建设。大力实施蓝天工程，推进城市热电联产项目、气化鞍山项目、重点企业污染治理及提标改造项目。推动实施净土工程，规划建设循环经济环保产业园，做好城市生活垃圾、餐厨垃圾、医疗垃圾、市政污泥、工业危废等的有效处理和资源再利用。

（五）进一步推进重点工作项目化，加速改革开放

以项目建设年为重点，全面实施重点工作项目化，在抓项目中深化改革，在上项目中扩大开放，进一步提高发展活力和竞争实力。

深入开展项目建设年活动。继续滚动实施“五个百日行动”，破除一切影响项目建设的障碍，做大做强一批已投产项目，开工建设一批已签约项目，积极推进一批已在谈项目，认真谋划一批牵动性项目。确保全年开工建设项目2700个，当年完成投资2100亿元。

不断扩大对外开放。把握国内外产业结构大调整大转移的机遇，实施更加积极主动的开放战略，重点引进一批优势工业集群项目、新兴服务业项目和牵动性强的大项目，推动开放朝着优化结构、拓展深度、提高效益方向转变。全年实际利用外资增长10%，引进域外资金增长20%，外贸出口增长12%。

加速综合配套改革。按照沈阳经济区综合配套改革试点，切实抓好土地供给，依法科学管理征而未批、批而未供、供而未用土地，做好棚户区和城中村土地征收征用工作，为项目建设提供更多发展空间；切实抓好融资服务，推动土地、房产等国有资产资本化，多渠道筹措资金，确保全年各类融资总量增长30%以上；继续抓好扩权强县区工作，推进城市管理重心下移，提高县（市）区发展的积极性和主动性；支持各级各类园区和新城新市镇在机构设置、绩效考评等方面进行体制机制创新。

落实人才强市战略。把人才作为推进改革开放、促进经济转型、提升创新驱动的关键要素。在政治待遇、生活待遇和事业发展等方面创造优惠政策，努力培养和吸引一批德才兼备、勇于创业、善于管理的各类人才队伍。

加强软环境建设。推进行政审批制度改革，主动解决项目企业在项目建设中遇到的难题，严格监督和严肃查处项目审批过程中的违纪违规行为，为经济发展营造一个效率高、服务好、讲诚信的良好环境。

大力发展非公有制经济。支持中小微企业发展，从资金扶持、企业减负、市场拓展、要素供给和高效服务等方面帮助企业解难题、渡难关。加快推进创新型中小企业培育工作。启动实施市长质量奖，加速培育知名品牌，提高民营企业核心竞争力，为民营企业转型升级创造良好环境。

（六）进一步推进以民生为重点的社会事业，建设幸福鞍山

大力发展各项社会事业，进一步推进城乡之间、各类人群之间基本公共服务均等化，继续为城乡群众办实事，在学有所教、劳有所得、病有所医、老有所养、住有所居、困有所助上取得新进展，让全市人民过上更加美好的生活。

千方百计扩大就业。强化职业技能培训，支持全民创业，做好大学毕业生、新生劳动力和就业困难群体的就业工作。全年实名制就业10.4万人，城镇登记失业率控制在3%以内，保持“零就业家庭”动态为零。

全面提升保障水平。加大社会保险征缴工作力度，扩大新型农村社会养老保险、城镇居民基本医疗保险覆盖面，提高各项社会保险和城乡低保标准。加快养老家园建设，推进社会养老事业和慈善事业发展。

加快回迁住房和保障性住房建设。续建回迁住房项目26项。新建廉租房、限价商品房等保障性住房8000套。推进城中村改造，解决城乡群众住房难题。

积极发展教育事业。新建扩建20所农村幼儿园，组建优质幼教集团。推进基础教育强县区工程。扩大优质高中覆盖面。建设中职、高职、应用本科相衔接的现代职业教育体系，创建全国职业教育示范园区。为辽宁科技大学、鞍山师范学院发展建设创造良好条件。

繁荣发展文化事业。加速公共文化服务体系全覆盖，新建7个县级和20个乡镇级电子阅览室，在每个社区和行政村规划建设文化广场。加快文艺院团改革与建设。完成有线电视数字化整转工作。建成2个国家级文化专业市场。

加速发展医疗卫生事业。建立基本保险、商业保险和政府救助相结合的医疗保障制度。在农村试行儿童白血病、先天性心脏病和肾病透析患者免费治疗。打造城区3—5公里急救半径绿色生命圈。实施市级公立医院药物统一采购和村卫生室基本药物制度。深化公立医院改革，推进海城公立医院改革试点。

努力发展体育事业。高标准建成奥体中心，做好十二届全运会鞍山赛区工作，办精彩全运，展鞍山风采。广泛开展全民健身系列活动，更新和新建100个城乡健身广场。

不断强化社会管理。继续做好受灾乡镇灾后重建工作，保证受灾群众生产生活。强化价格调控监管，增加困难群体生活补贴。推进“六五”普法，建设法治城市。加强食品安全风险监测，严厉打击生产销售假冒伪劣食品药品等违法犯罪行为。完善立体化社会治安防控体系，加大信访问题化解力度，加强和创新社会管理，创造和谐稳定的社会环境。强化安全生产和交通、消防安全，坚决遏制重特大安全事故发生。

进一步做好广播电视、新闻出版、人口与计划生育、妇女儿童、老龄、残联、民族、宗教、外事、侨务、对台、统计、史志、档案、气象、地震、人防、民兵预备役等工作。

（七）进一步加强自身建设，打造人民满意政府

把人民对美好生活的向往，作为各级政府矢志不渝的工作目标，努力打造让人民满意的法治型、服务型、实干型、创新型政府。

建设法治型政府。自觉接受人大及其常委会法律监督、工作监督和政协民主监督，认真办理好人大代表议案、建议和政协提案，广泛听取各民主党派、工商联和无党派人士的意见和建议。支持检法机关依法履行职责。把公众参与、专家论证、集体讨论作为行政决策的必要程序，不断提高依法决策、科学决策、民主决策水平。

建设服务型政府。牢固树立亲民为民理念，努力办好顺民意、解民忧、惠民生的好事实事，解决好事关民生的难事急事。牢固树立亲商爱商理念，察企业之所难，帮企业之所需。健全权力运行和监督体系，深入开展政风行风民主评议，发挥好公共资源交易中心、政府门户网站等服务平台作用，自觉接受人民和社会监督，做到干部清正、政府清廉。

建设实干型政府。以重点工作项目化为核心，深入实施项目法人制、协调推进制和决策执行制。完善责任体系，强化绩效考评，实行问责追究，提高工作效率。进一步改进文风会风，开短会、讲短话、干实事，全面提升政府执行力、公信力。

建设创新型政府。把改革创新精神贯穿到政府工作全过程，冲破一切妨碍发展的思想观念，改变一切束缚发展的做法和规定，革除一切影响发展的体制弊端，推进各项工作不断取得新成绩。

各位代表、同志们，新的目标催人奋进，新的征程任重道远。让我们在党的十八大精神指引下，在省委、省政府和市委的正确领导下，凝心聚力，克难攻坚，真抓实干，为建设富庶文明幸福新鞍山，率先建成小康社会而努力奋斗！
